# Calcium influx-mediated translocation of *m*-calpain induces Ku80 cleavage and enhances the Ku80-related DNA repair pathway

**DOI:** 10.18632/oncotarget.8791

**Published:** 2016-04-18

**Authors:** Kyung Hye Baek, Han Vit Yu, Eosu Kim, Younghwa Na, Youngjoo Kwon

**Affiliations:** ^1^ College of Pharmacy, Graduate School of Pharmaceutical Sciences, Ewha Womans University, Seoul, Republic of Korea; ^2^ Department of Psychiatry, Yonsei University College of Medicine, Seoul, Republic of Korea; ^3^ College of Pharmacy, CHA University, Pocheon, Republic of Korea

**Keywords:** m-calpain, Ku80-related DNA repair, adriamycin, DNA damage

## Abstract

Proteomic analysis of ionomycin-treated and untreated mammary epithelial MCF10A cells elucidated differences in Ku80 cleavage. Ku80, a subunit of the Ku protein complex, is an initiator of the non-homologous, end-joining (NHEJ), double-strand breaks (DSBs) repair pathway. The nuclear Ku80 was cleaved in a calcium concentration-dependent manner by *m*-calpain but not by *m*-calpain. The cleavage of nuclear Ku80 at its α/β domain was validated by Western blotting analysis using flag-tagged expression vectors of truncated versions of Ku80 and a flag antibody and was confirmed in *m*-calpain knock-down cells and *in vitro* cell-free evaluation with recombinant proteins of calpains, Ku70, and Ku80. In addition, the cleaved Ku80 still formed a Ku heterodimer and promoted DNA DSB repair activity. Taken together, these findings indicate that translocated *m*-calpain enhances the NHEJ pathway through the cleavage of Ku80. Based on the present study, *m*-calpain in DNA repair pathways might be a novel anticancer drug target, or its mechanism might be a possible route for resistance acquisition of DNA damage-inducing chemotherapeutics.

## INTRODUCTION

There are many ways to treat cancers such as interrupting cell division, targeting angiogenesis or growth factors, and inhibiting synthesis of nucleic acids. Induction of DNA damage and interference with DNA repair is one of the preferred ways to prevent the proliferation of cancer cells [1]. DNA can be damaged by endogenous oxidative stress or exogenous toxic agents such as radiomimetic drugs, ionizing radiation (IR), or ultraviolet (UV) light [2–5]. When damaged, DNA might undergo some chemical alterations such as strand breaking, crosslinking, and the loss of bases [1]. Among these types of DNA damage, double-strand breaks (DSBs) are the most severe [1–5] due to the resulting reduction in genomic stability, induction of chromosomal aberration, and loss of genetic information, which eventually leads to cell death [6, 7]. In eukaryotes, there are two major DNA DSB repair pathways, the homologous recombination (HR) pathway and the non-homologous end-joining (NHEJ) pathway [8]. The most important difference between the two pathways is cell-cycle dependency. HR is cell-cycle specific because homologous DNA segments exist only after DNA replication; therefore, HR has a low probability of producing errors [9–12].

NHEJ is the main pathway in the repair of DSBs because it repairs the DNA DSBs by ligating both ends of the broken DNA. This pathway is less accurate than HR, but it can operate throughout all phases of the cell cycle [13, 14]. The initiator of NHEJ is the Ku complex, which recognizes DNA DSB ends. The Ku complex exists in mammalian cells but not in yeast and is a heterodimer composed of two subunits, Ku70 (70 kDa) and Ku80 (80 kDa) [15]. Ku proteins play an important role in numerous cellular processes including V(D)J (Variable-(Diversity)-Joining) recombination, transcription, regulation of heat shock proteins, and telomere maintenance [16, 17]. Furthermore, many reports have suggested that Ku proteins have positive effects on the progression of cancer cells. If Ku is deficient in cells, the aforementioned cellular processes cannot be performed properly, and cells undergo tumorigenesis [17–19]. After DNA damage occurs, the Ku complex binds to DNA break ends and recruits NHEJ proteins such as DNA protein kinase (DNA-PK) [20]. Recent studies have shown that inhibition of the extracellular signal-regulated kinases (ERK) pathway enhances the NHEJ pathway through the activation of DNA-PKcs [21, 22].

Although the induction of DNA damage is the main treatment for cancer, it can promote carcinogenesis by damaging normal cells, to which the majority of the adverse effects of anticancer therapy are attributed. Moreover, the DNA process is easily affected by cell cycle-related proteins and apoptosis regulators [23, 24]; therefore, it is very important to control DNA damage and to repair it properly in order to minimize side effects.

Recent studies have suggested that calpains are one of these DNA process-regulating proteins because it has been demonstrated that *m*-calpain but not *μ*-calpain can translocate from the cytosol to the nucleus. Translocated *m*-calpain proteolyzes topoisomerase I, which produces DNA topological problems during the cell cycle. The relaxation activity of topoisomerase I is greater when it is truncated by *m*-calpain [24]. Calpains belong to the family of calcium-dependent cytoplasmic cysteine proteases; due to the high calcium level in cancer cells resulting from the low buffering system, calpains are easily activated to process toward survival [25–29]. Among 15 human calpain isoforms, calpains 1 (*μ*-calpain) and 2 (*m*-calpain), named after the respective micromolar and millimolar calcium concentrations required for their full activation *in vitro*, are expressed ubiquitously and have been the most intensively studied [30, 31]. Calpain uses various proteins as substrates; therefore, we attempted to classify the substrates of *m*-calpain in order to understand its diverse roles. First, calpain regulates transcription through the proteolysis of transcription factors such as nucleolin, IkBa, and histone 2B [32–34]. Second, calpain has effects on cell motility and adhesion by cleaving cytoskeletal proteins such as spectrin, tubulin, and filamin [35–45]. In addition, there are many signaling proteins and apoptosis regulators such as apoptosis-inducing factor (AIF), Bax, Bid, and caspases that act as calpain substrates, suggesting that calpain is likely involved in apoptosis and cellular survival [35, 46–50]. In addition, it is also involved in angiogenesis by altering blood coagulation proteins such as fibrinogen and kininogen [51] (Tables S1-S3 in Supplementary Information).

Although not fully understood, it seems very important to define the unique roles of each calpain isoform, because where and which calpain is overexpressed can differ according to cancer type. For instance, both *μ*- and *m*-calpain are closely involved in the invasion of lung cancer. However, it has also been shown that *m*-calpain is more involved in the invasion of prostate cancer than is *μ*-calpain [52, 53]. Furthermore, *μ*-calpain plays a larger role in the apoptosis and regression of blood vessels than does *m*-calpain, whereas *m*-calpain has a larger effect on migration and angiogenesis [54, 55]. Thus, demonstrating their specific roles could be a key factor in the development of effective anti-cancer drugs.

In the present study, we hypothesized that translocated *m*-calpain affects DNA processes such as replication, damage, and repair based on a literature search (Tables S1-S3). We found that *m*-calpain translocated as the result of calcium influx was involved in DNA DSB repair, especially in the NHEJ pathway through proteolysis of nuclear Ku80 but not Ku70. We also elucidated that cleaved Ku80 was still able to form a heterodimer with Ku70 and enhance DNA repair activity. Taken together, these results suggest that the mechanism of *m*-calpain in DNA repair pathways might be used as a novel anticancer drug target or a possible route for resistance acquisition of DNA damage-inducing chemotherapeutics.

## RESULTS

### Calcium influx-mediated translocation of *m*-calpain from the cytosol to the nucleus attenuates ERK in MCF10A cells

**Figure 1:**
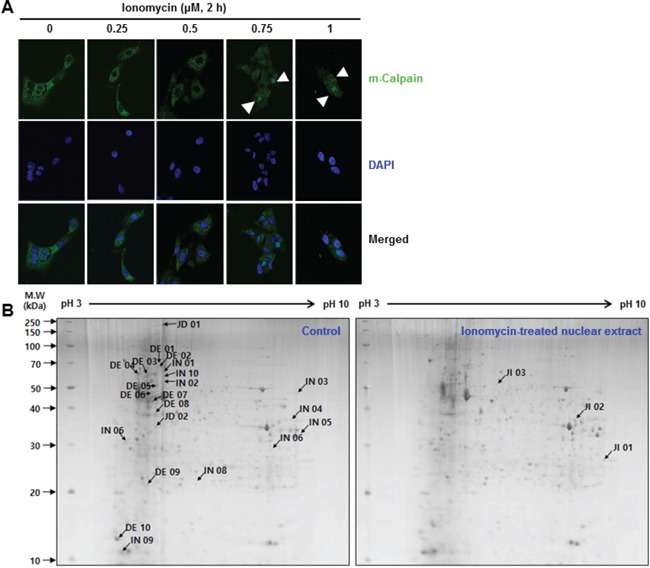
**A.** Cytosolic *m*-calpain was translocated to the nucleus by Ca^2+^ influx in MCF10A cells. An immunofluorescence assay using confocal microscopy revealed that ionomycin treatment led to translocation of cytosolic *m*-calpain to the nucleus. The nucleus was stained with DAPI, and *m*-calpain was indicated by an Alexa Fluor^®^ 488-conjugated secondary antibody. **B.** Changes upon ionomycin treatment of MCF10A cells were analyzed by 2-DE and MS. Nuclear extracts of MCF10A cells, after treatment with or without 1 μM ionomycin for 2 hours, were prepared by the cellular fractionation method. Following electrophoresis, gels were stained with Coomassie Blue G-250.

Immunofluorescence analysis confirmed that translocation of the cytosolic protein *m*-calpain to the nucleus was induced by calcium influx (Figure 1A). *m*-Calpain was located in the cytosol in untreated MCF10A cells. While it clearly translocated from the cytosol to the nucleus when treated for 2 hours with an ionomycin concentration of 0.75 μM or higher. Ionomyci, an ionophore, was used to increase the level of intracellular calcium. Thus, activated *m*-calpain can affect nuclear proteins involved in cellular processes occurring in the nucleus such as DNA replication, repair, and transcription (Tables S1-S3). To verify the effect of Ca^2+^ influx-mediated translocated *m*-calpain on nuclear proteins, nuclear extracts of ionomycin-treated and untreated cells were prepared and analyzed using two-dimensional gel electrophoresis (2-DE). In comparison with untreated MCF10A cells, intensities of 10 DE-labeled spots of the treated cells were decreased more than two-fold, and two spots labeled JD were no longer present. In addition, 10 spots were intensified more than two-fold and were thus named IN spots. The three newly appeared spots were labeled JI (Figure 1B). These spots changed dramatically upon treatment with 1 μM ionomycin for 2 hours and were further analyzed by mass spectrometry (MS). The results are summarized in Tables 1–3. As we expected, cytoskeletal proteins such as talin, actin, and filamin were shown to be the most influenced by calcium influx, because calpain cleaves a substantial number of such cytoskeletal and structural proteins (Table S1). Moreover, the expressions of heat shock proteins and RNA-binding proteins such as ribonucleoprotein K were changed with the calcium level. Among these proteins, we focused on large ERK kinase in the DE03 spot. As mentioned earlier, ERK kinase has been reported to be involved in the DNA repair pathway by enhancing the NHEJ pathway [21, 22]. Taken together, these results led us to hypothesize that the DNA repair pathway, especially the NHEJ pathway, is affected by change in intracellular calcium level.

**Table 1:** Expected proteins of labeled DE spots whose intensity decreased more than two-fold compared with the control in Figure 1B according to MS analysis

Spot	Protein name	Spot	Protein name
DE01	MTHSP75	DE06	Actin, spectrin
	Chaperonin (HSP60)		Lamin B1
	Lamin isoform C		Keratin 1
DE02	Tumor necrosis factor type 1 receptor-associated protein		Rho guanine nucleotide exchange factor 33
	60kDa heat shock protein	DE07	Lamin-binding protein
	Pyruvate kinase		Cytokeratin
	Keratin 1	DE08	Lamin-B1 isoform 1
DE03	Chaperonin (HSP60)		Annexin A2 isform 2
	Heat shock protein gp96 precursor		Glyceraldehyde-3-phosphate dehydrogenase
	Large ERK kinase	DE09	HP1Hs-γ
	Heterogeneous nuclear ribonucleoprotein K		Heterogeneous nuclear ribonucleoprotein K
DE04	Cytokeratin 9		Hp1h2-γ
	Heat shock protein	DE10	Apolipoprotein B mRNA editing enzyme
DE05	β-actin		Cytokeratin 2
	cytokeratin 2		Acidic ribosomal protein P1

**Table 2:** Expected proteins of labeled IN spots whose intensity increased more than two-fold compared with the control in Figure 1B according to MS analysis

Spot	Protein name	Spot	Protein name
IN01	Actin	IN06	Amphiphysin II
IN02	Actin		SETD 8 protein
IN03	Cytokeratin 2,9	IN07	Ankyrin repeat domain
	Translation elongation factor		p 107
	Plasminogen	IN08	PNAS-102
	Apolipoprotein B mRNA editing enzyme		Heterogeneous nuclear ribonucleoprotein H
IN04	MEGF5		Glutathione peroxidase
	Cerebrin 30	IN09	Unnamed protein
	Cytokeratin 9	IN10	Actin, talin
IN05	Cytokeratin 9		POTE ankyrin domain family

**Table 3:** Expected proteins of spots that were newly induced (JI) or disappeared (JD) upon treatment with ionomycin compared with the control in Figure 1B according to MS analysis

Spot	Protein name	Spot	Protein name
JI01	Unnamed protein	JD01	actin
JI02	Annexin A2		POTE ankyrin domain family
	Aldolase A		Filamin A
	Fructose bisphosphate aldolase		Plectin 1, imtermediate filament binding protein
JI03	Actin-related protein 3	JD02	ES130
	Brain-specific angiogenesis inhibitor-associated protein		Serine/threonine-protein phosphatase PP1-β catalytic subunit

### Ionomycin-induced cleavage of nuclear Ku80 in MCF10A cells is blocked by calpeptin, a calpain inhibitor

**Figure 2:**
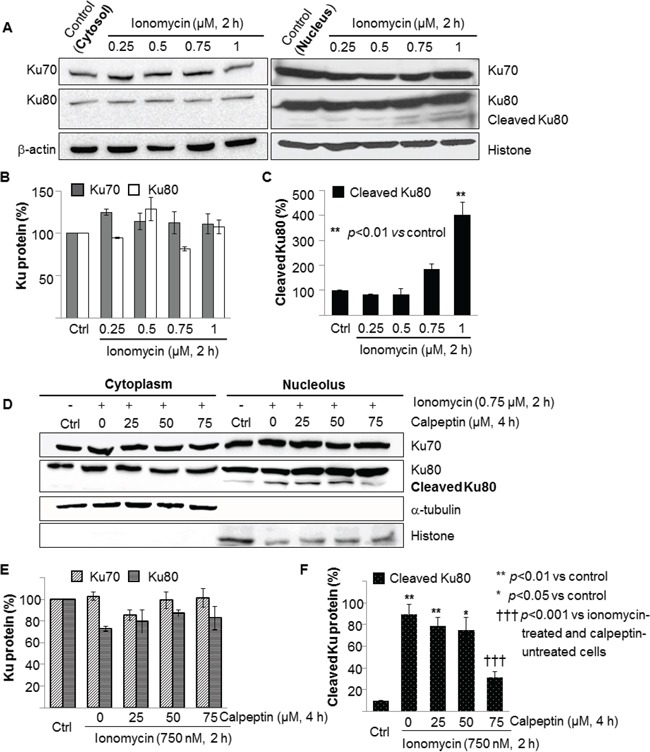
Ionomycin-induced cleavage of nuclear Ku80 but not cytosolic Ku80 in MCF10A cells was attributed to activated calpain **A.** MCF10A cells treated with or without ionomycin at designated concentrations for 2 hours were lysed and prepared for cytoplasm and nucleoplasm separately, followed by Western blotting. Cytosolic Ku70 and Ku80 and nuclear Ku70 were not influenced by ionomycin-induced calcium influx. However, nuclear Ku80 was cleaved in a calcium concentration-dependent manner. The levels of Ku70, Ku80, and cleaved Ku80 proteins in **A.** were normalized to the β-actin or α-tubulin content for cytoplasm and the histone content for nucleoplasm and depicted as histograms in **B.** and **C.** Values represents the mean ± SD of three independent experiments. **p<0.01 *vs.* the values of ionomycin-untreated MCF10A cells. **D.** Calpeptin, a calpain inhibitor, blocked cleavage of nuclear Ku80 in a concentration-dependent manner, indicating that translocated *m*-calpain induced proteolysis of nuclear Ku80. The levels of Ku70, Ku80, and cleaved Ku80 proteins in (D) were normalized using the same method as mentioned above. **E, F.** Bars represent the mean ± SD of three independent experiments. *p<0.05, **p<0.01 *vs.* the values of ionomycin-untreated MCF10A cells. †††*p*<0.001 *vs.* the values of ionomycin-treated and calpeptin-untreated MCF10A cells.

Our observation of ERK attenuation induced by abnormally increased levels of intracellular calcium (Figure 1B and Table 1), in combination with a literature study of the relationship between ERK and NHEJ, led us to hypothesize that NHEJ is enhanced by calcium influx [21]. Further, previous studies have shown that the expression of Ku proteins, the initiators of the NHEJ pathway, were altered by oxidative stress in the nucleus but not in the cytosol, and that Ku80 in particular was cleaved by some proteases [56, 57]. Therefore, we decided to further clarify the effects of calcium influx on Ku70 and Ku80 by identifying their expression levels in the cytosol and nucleus. As shown in Figure 2A and 2B, the expression levels of Ku70 and Ku80 were not affected by calcium influx in the cytosol, nor was that of nuclear Ku70. However, Ku80 in the nucleus was proteolyzed by calcium influx in a calcium concentration-dependent manner (Figure 2C). This data supports the notion that Ku80 is cleaved by enzymes activated by calcium.

To verify the identity of the enzyme that cleaved nuclear Ku80, we treated the cells with calpeptin prior to treatment with 0.75 μM ionomycin since a previous study had reported that *m*-calpain was translocated from the cytosol to the nucleus after activation by calcium [24]. Calpeptin is a well-known, cell-permeable calpain inhibitor and synthetic peptidomimetic aldehyde moiety-conteining molecule. The calpain inhibitory activity of calpeptin was reported as 0.04 μM of the IC_50_ value in platelets [58]. As shown in Figure 2D, cytosolic Ku70 and Ku80 and nuclear Ku70 were not changed by co-treatment with ionomycin and calpeptin. However, nuclear Ku80 was cleaved upon treatment with ionomycin, as seen in Figure 2A, and the extent of proteolysis was decreased by calpeptin treatment in a concentration-dependent manner. This data suggests that calpain is the main enzyme causing cleavage of nuclear Ku80.

### Recombinant *m*-calpain cleaves only Ku80, not Ku70, in the presence of calcium

**Figure 3:**
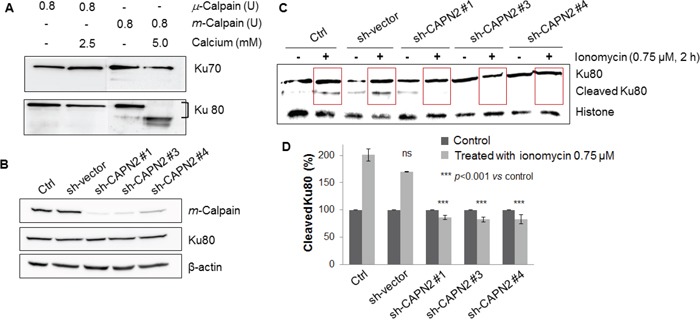
**A.** Purified *m*-calpain cleaved only Ku80 not Ku70 in the presence of calcium. After incubation with purified calpains and Ku proteins in calpain assay buffer, Western blotting analysis was performed using anti-Ku70 (upper panel) and anti-Ku80 (lower panel) antibodies. *m*-Calpain showed specific proteolytic activity toward Ku80. **B.** The expression of *m*-calpain in MCF10A cells was knocked down by short hairpin RNA transfection without any influence on the expression on Ku80. **C.** Cleavage of Ku80 was not observed in sh-CAPN2-infected MCF10A cells. **D.** The levels of proteins in (C) were normalized to the histone content of ionomycin-untreated cells (control) and depicted as histograms. Bars represent the mean ± SD of three independent experiments. *** *p*<0.001 *vs.* the values of ionomycin-untreated MCF10A cells.

Recombinant *μ*- and *m*-calpains were activated with calcium in calpain assay buffer and reacted with recombinant Ku70 and Ku80 as substrates. After performing cell-free assays, it was evident that Ku70 was not the substrate of either *μ*-calpain or *m*-calpain. Ku80, however, was proteolyzed by *m*-calpain but not by *μ*-calpain (Figure 3A). Therefore, it is clear that *m*-calpain cleaves only Ku80, and that Ku80 is the specific substrate of *m*-calpain.

### Nuclear Ku80 is not cleaved in *m*-calpain knock-down MCF10A cells

After confirmation that proteolysis of Ku80 was induced by only *m*-calpain using purified recombinant proteins, we attempted to validate this result in cells by performing an *m*-calpain-targeted short hairpin RNA (sh-CAPN2) experiment. We infected the cells with four sh-CAPN2 plasmids, which underwent selection with puromycin for two weeks. Among the four sh-CAPN2s, only three succeeded to knockdown *m*-calpain, and the level of Ku80 was not influenced (Figure 3B). Following investigation of the downregulation of the *m*-calpain level, we induced calcium influx with 0.75 μM ionomycin in sh-vector-infected and sh-CAPN2-infected MCF10A cells. Compared with ionomycin-untreated cells, proteolysis of Ku80 was increased in the control and sh-vector-infected MCF10A cells; however, there were no changes in the sh-CAPN2#1-, sh-CAPN2#3-, or sh-CAPN2#4-infected cells. These results confirm that *m*-calpain is the key enzyme causing the proteolysis of nuclear Ku80 upon calcium influx.

### *m*-Calpain cleaves the α/β domain of Ku80

**Figure 4:**
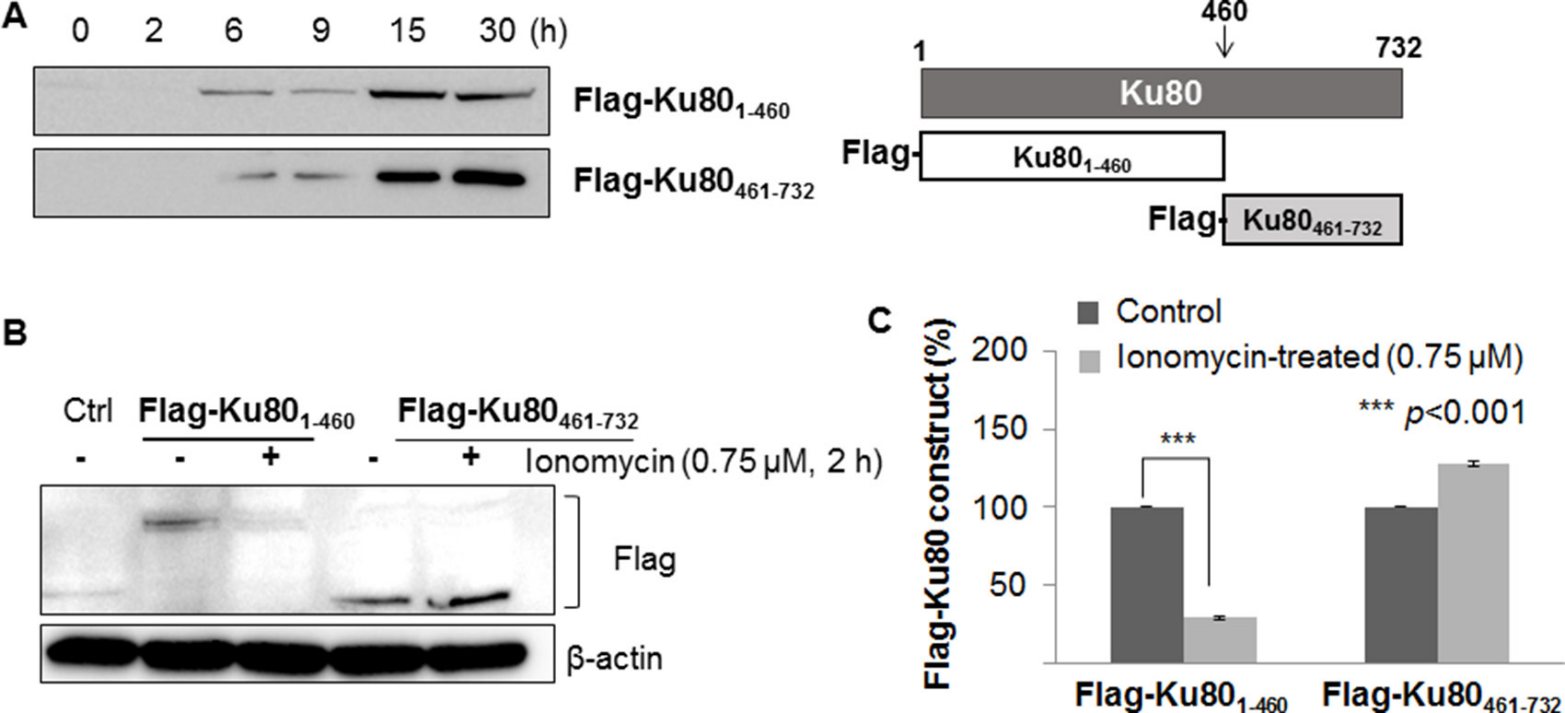
*m*-Calpain cleaved the α/β domain located at the N-terminus of Ku80 **A.** To elucidate the cleavage region, MCF10A cells were infected with Flag-Ku80_1-460_ (N-terminal) and Flag-Ku80_461-732_ (C-terminal) constructs. Western blotting analysis was performed with an anti-flag antibody to confirm transfection and expression of flag-tagged C- and N-terminal Ku80 proteins. These proteins were fully expressed after 15 hours of transfection. **B.** Activated *m*-calpain by ionomycin treatment cleaved the N-terminal region of Ku80. **C.** The levels of proteins in (B) were normalized to the β-actin content of ionomycin-untreated cells (control) and depicted as histograms. Bars represent the mean ± SD of three independent experiments. ***p<0.001 *vs.* the values of ionomycin-untreated MCF10A cells.

The 293FT cells separately transfected with the α/β domain (N-terminus of Ku80) and the carboxy helical domain (C-terminus of Ku80) were analyzed following incubation with or without ionomycin to verify the cleavage region of Ku80. Prior to treatment with ionomycin, a 15 hour transfection was set as the most efficient time by monitoring the extent of protein expression encoded by the transfected genes with an anti-flag antibody (Figure 4A). Figure 4B shows that the carboxy domain of Ku80 was not altered by calcium influx; however, the α/β domain of Ku80 was diminished by calcium influx. These data demonstrate that the cleavage site of Ku80 for *m*-calpain is located in the N-terminal region of Ku80, called the α/β domain.

### Cleaved Ku80 forms a heterodimer with Ku70

**Figure 5:**
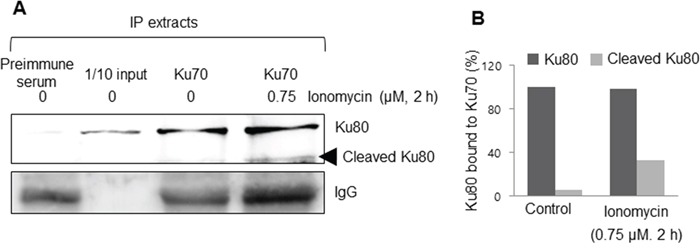
Cleaved Ku80 formed a heterodimer with Ku70 **A.** An immunoprecipitation assay showed that Ku70 could bind to both the full-length and cleaved Ku80, meaning that *m*-calpain-mediated cleaved Ku80 can still form a Ku complex. **B.** The percentage content of bound K80 to K70 is depicted as a histogram.

In order to investigate the function of cleaved Ku80, immunoprecipitation was performed. Based on previous reports that Ku70 and Ku80 form a heterodimer, a Ku70 antibody was used to precipitate the immune complex, and Western blotting was performed with a Ku80 antibody. In untreated cells, one band of approximately 80 kDa was detected, corresponding to full-length Ku80. However, there were two bands in the ionomycin-treated MCF10A cell immunoprecipitates, with the sizes of the detected proteins matching the sizes of the cleavage products of Ku80 shown in previous studies (Figure 5). Therefore, it was concluded that cleaved Ku80 can dimerize with Ku70 in the same manner as full-length Ku80.

### Cleaved Ku80 enhances the repair activity of adriamycin-mediated DNA damage

**Figure 6:**
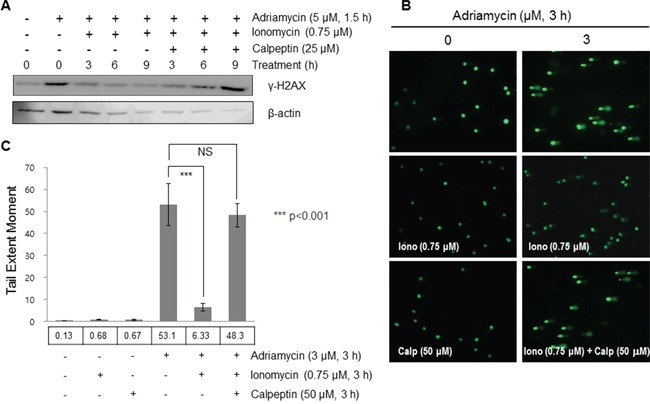
Cleaved Ku80 enhanced the repair activity of DNA damaged caused by adriamycin **A.** MCF10A cells were treated with 5 μM of adriamycin for 1.5 hours, followed by treatment alone or combined with 0.75 μM ionomycin and 50 μM of calpeptin in fresh media for the designated times. Cell lysates were then subjected to Western blotting. **B.** A comet assay was performed to evaluate the effect of cleaved Ku80 on DNA repair following treatment of ionomycin, calpeptin, and adriamycin alone or co-treatment of ionomycin-adriamycin and ionomycin-calpeptin-adriamycin. Tail extent was obtained with the lengths of the tail and head by analysis of images using the Komet software. **C.** Columns and error bars indicate the mean ± SD (n = 50). ***p<0.001 shows a significant difference from the value of adriamycin-treated cells. Adriamycin was used to cause DNA damage, and γ-H2AX was used as a marker of DNA damage. DNA damage caused by adriamycin was more efficiently repaired under *m*-calpain activation conditions than under suppressed conditions.

Although cleaved Ku80 was shown to form a heterodimer with Ku70, additional experiments were performed to determine its effects on DNA repair activity. In order to identify the biological implications of *m*-calpain-mediated cleavage of Ku80 on DNA repair, we induced DNA damage with adriamycin treatment and then activated *m*-calpain with ionomycin treatment in order to cleave Ku80. In addition, we treated the cells with calpeptin to inhibit *m*-calpain activation. Firstly, the expression of γ-H2AX was observed at 3, 6, and 9 hours after *m*-calpain activation. When mammalian cells undergo DNA damage, histones are phosphorylated to γ-H2AX, a widely used marker of DNA damage [59]. As shown in Figure 6A, the expression of γ-H2AX was increased upon treatment with adriamycin, and the extent of this increased expression was decreased by treatment with ionomycin. However, the increased expression level of γ-H2AX by adriamycin was maintained upon treatment with calpeptin. These results imply that *m*-calpain, activated by calcium influx, ameliorates DNA repair and decreases adriamycin efficacy. In addition, a comet assay was performed to identify the extent of the DNA damage. The comet assay is a sensitive method for the detection of DNA damage in individual cells [60]. The extent of the tail formation is regarded as the extent of DNA damage. As shown in Figure 6B and 6C, neither ionomycin nor calpeptin alone induced DNA damage, while treatment with 3 μM adriamycin induced a comet tail. A comet tail was rarely seen in combined treatment with ionomycin and adriamycin in MCF10A cells. When calpeptin was co-treated with adriamycin and ionomycin, the comet tail was restored to a similar extent to that induced by adriamycin. These results confirm the positive effect of *m*-calpain-mediated Ku80 cleavage on DNA repair activity.

## DISCUSSION

It is known that *μ*- and *m*-calpains participate in several cellular processes such as transcription, apoptosis, maintenance of cellular structure, and carcinogenesis [29–31, 52]. The proteolytic activity of *μ*- and *m*-calpains against diverse substrate proteins must be tightly controlled, as their abnormal activation has been implicated in the pathogenesis of many diseases such as Alzheimer's disease, muscular dysfunction, myocardial infarct, and tumor progression [26–31, 35, 47–49, 53, 55]. However, calpain isotype-specific cleavage of nuclear proteins and its biological importance and roles in the pathogenesis associated with diseases remain incompletely investigated. The insufficient or imprecise understanding of calpain isotype-specific functions might be due to the structural similarity of substrate-binding catalytic sites in *μ-* and *m*-calpains and a lack of stable and potent isotype-specific inhibitors available for *in vivo* experiments [30, 61]. We first attempted to summarize a plethora of proteins that are cleaved by *m*-calpain in order to better understand the isoform-specific biological implications of *m*-calpain. As listed in Tables S1-S3, translocated *m*-calpain affects DNA processes including replication, damage, and repair; however, no substrate related to DSBs repair has been reported. It has however been reported that calcium influx-activated topoisomerase I proteolysis is caused by *m*-calpain translocation. Moreover, truncated topoisomerase I exhibited an enhancement of DNA relaxation activity compared with its full length, leading to a reduction in camptothecin efficacy related to cell death [24]. In this regard, the current study focused on the role of *m*-calpain in DNA DSB repair. We found that ionomycin-activated and translocated *m*-calpain induced substantial abatement in the expression level of ERK kinase, as seen by 2D-gel and MS analysis. Based on reports that the inhibition of ERK kinase activation enhanced the NHEJ pathway of DNA DSB repair through DNA-PKcs activation [21], and that Ku proteins were downregulated and cleaved by proteases under oxidative stress conditions [56, 57], we evaluated the influence of ionomycin-activated *m*-calpain nuclear translocation on the NHEJ pathway of DNA DSB repair through investigation of NHEJ pathway-related proteins. Ionomycin treatment activated and translocated cytosolic *m*-calpain to the nucleus (Figure 1A). Translocated *m*-calpain cleaved nuclear Ku80 but not Ku 70 (Figure 2A & 2C). Calpeptin, a calpain inhibitor, blocked the cleavage of nuclear Ku80 (Figure 2D & 2F), which was confirmed in *m*-calpain knockdown cells and in *in vitro* cell-free *μ*- and *m*-calpain assays with purified recombinant calpains and Ku proteins (Figure 3). This is the first report of Ku80 as the specific substrate for *m*-calpain. We further determined the cleavage region of Ku80 to be its α/β domain (Figure 4). When DSBs are caused by various DNA-damaging agents such as IR, chemotherapeutics, UV light, and radiotherapy, Ku70 and Ku80 form a heterodimer [18] and initiate the NHEJ pathway by binding to both ends of DSBs, followed by recruitment of other repair proteins such as DNA-PK, XRCC4, and ligase IV [10, 11, 13, 14]. Ku proteins consist of three subunits, an α/β domain, a β-barrel domain, and a helical carboxy arm. The β-barrel domain, the central part of Ku proteins, is involved in non-specific binding to the DNA backbone. The α/β domain is at the N-terminus of Ku proteins, and the helical C-terminal arm is related to the binding to repair proteins. In particular, the C-terminus of Ku proteins is involved in dimerization and association with DNA-PKcs [18]. Therefore, cleavage of the N-terminus of Ku80, caused by activated *m*-calpain in the nucleus, does not interfere with the ability of Ku80 to form dimers with Ku70, as observed from the immunoprecipitation analysis shown in Figure 5. Although the extent of nuclear cleaved Ku80 by calcium influx-activated *m*-calpain was not as high as that of intact full-length nuclear *m*-calpain (Figure 2), nuclear cleaved Ku80 reduced the adriamycin-induced DNA DSBs (Figure 6). Previously, it has been reported that Ku80 (732 aa) is cleaved at the Asp-730 residue by caspase-3 during apoptosis in Jurkat cells [62]. This caspase-3-mediated C-terminal cleavage prevented Ku80 from associating with DNA-PKcs because the C-terminus of Ku80 is the binding site for DNA-PKcs. The repair of DNA DSBs was eventually retarded during apoptosis [63]. The C-terminal last two-residue cleaved form of Ku80 is distributed in the cytoplasm of apoptotic cells, and the localization of full-length Ku80 depends on the cell cycle. The full-length form of Ku80 localizes in the nucleus in interphase, in the cytoplasm during mitosis, and in the nucleus during late telophase/early G1 phase [63, 64]. The Ku80 cleavage site and localization seem to be very important for its DNA repair function. The *m*-calpain-mediated Ku80 N-terminal cleavage occurred and its cleaved form was retained in the nucleus, while the Ku80 C-terminal-truncated by caspase-3 was localized in the cytoplasm.

Improper repair of DBSs, the most lethal form of DNA damage, can lead to chromosomal aberrations, genetic instability, and cellular death [1–5]. NHEJ joins the ends of DSBs regardless of the cell-cycle, indicating that NHEJ is the most predominant DSB repair pathway, especially for damage caused by IR and radiomimetic drugs [1, 11, 12]. Therefore, the role of *m*-calpain in NHEJ DNA repair through the cleavage of Ku80 in the nucleus has to be cautiously considered during treatment of DNA damage caused by anticancer drugs such as the topoisomerase II-targeting drugs, adriamycin, etoposide, and quinolones [65]. Adriamycin is considered to be the most effective anticancer drugs in clinical use; however, resistance is common, demonstrating a main disablement to successful treatment. It has been previously reported that adriamycin increased the intracellular calcium concentration and ROS generation in rat cardiomyocytes [66]. A higher calcium level has been observed in adriamycin-resistant leukemia cells compared with adriamycin-sensitive cells; however, the correlations between intracellular free calcium concentration and calmodulin content with induction of drug resistance in leukemia cells have not been clearly defined [67]. Treatment with cepharanthine, a calcium antagonist, at nontoxic concentrations moderately enhanced adriamycin cytotoxicity against sensitive K562 and Ov2780 human cancer cells, while a significant enhancement was observed when resistant K562/ADM and AD 10 human tumor cells were co-treated with cepharanthine and adriamycin [68]. The results obtained in the current study, showing that activation and translocation of *m*-calpain induced by calcium influx cleaved nuclear Ku80 and reduced adriamycin cytotoxicity, suggest a link between adriamycin resistance and the high level of intracellular calcium concentration in cells. The mechanism of drug resistance is extremely complicated; therefore, our findings are not a solid resolution to resistance. However, DNA damage and repair are closely related to carcinogenesis, and our proposed mechanism will be a novel way to approach cancer therapy through the inhibition of calpain activation in combination with radiomimetic chemotherapeutics.

## MATERIALS AND METHODS

### Materials

Dimethyl sulfoxide (DMSO), ethylene glycol tetraacetic acid (EGTA), β-mercaptoethanol, CaCl_2_, MgCl_2_, NaOH, Triton X-100, ionomycin, NP-40, sodium deoxycholate, ammonium persulfate, KCl, formaldehyde, puromycin, penicillin, bromophenol blue, and ethanol were purchased from Sigma (USA). Phenylmethylsulfonyl fluoride (PMSF) was obtained from Bioworld (USA); and protease inhibitor cocktail, bovine serum albumin (BSA), and acrylamide solution were from GenDEPOT (USA). Sodium chloride (NaCl), ethylenediaminetetraacetic acid (EDTA), sodium dodecyl sulfate (SDS), 4-(2-hydroxyethyl)-1-piperazineethanesulfonic acid (HEPES), and glycine were purchased from Duchefa (The Netherlands), and Tween 20 was obtained from Junsei Chemical Co., Ltd (Japan). Tris-HCl and glycerol were purchased from USB (USA).

### Cell cultures

MCF10A, a human breast epithelial cell line (ATCC CRL-10317), was purchased from the Korean Cell Line Bank (Seoul, Korea). HEK293FT (referred to as 239FT), a human embryonic kidney cell line (ATCC PTA-5077), was kindly provided by Prof. H.G. Yoon (Yonsei University, Korea). MCF10A cells were grown in Dulbecco's Modified Eagle's Medium (DMEM, Welgene, Korea) supplemented with Nutrient Mixture F-12 (DMEM/F-12, Invitrogen, CA), 10% horse serum, 1% penicillin, 20 ng/mL of EGF, 0.5 mg/mL of hydrocortisone, 100 ng/mL of cholera toxin, and 10 μg/mL of insulin. 293FT cells were grown in DMEM supplemented with 10% fetal bovine serum (FBS, Hyclone laboratories Inc. USA) and 5% penicillin. Both cell lines were grown in a humidified 5% CO_2_ incubator at 37°C. All media were changed every 2-3 days, and cells were subcultured at 1:5 to 1:10.

### Nuclear fractionation

MCF10A cells were harvested by centrifugation at 3200 rpm and room temperature for 3 minutes, and the pellet was washed with phosphate buffered saline (PBS) in the same manner. The pellet was subsequently resuspended in 200 μL of buffer A (10 mM HEPES, 10 mM KCl, 0.1 mM EDTA, 1 mM dithiothreitol (DTT), and 0.5 mM PMSF adjusted to pH 7.5 with HCl) and incubated on ice for 10 minutes. Then, 10% NP-40 was added and vortexed for 10 seconds. The supernatant was collected following centrifugation for 10 minutes at 12000 rpm and 4°C, and the pellet was washed once with buffer A in the same manner and resuspended in 50 μL of buffer B (20 mM HEPES, 0.4 M NaCl, 1 mM EDTA, and 1 mM PMSF adjusted to pH 7.9 with HCl). Following incubation on ice for 30 minutes, the supernatant was collected by 10-minute centrifugation at 12,000 rpm and 4°C. The supernatant was the nuclear extract and was stored at −20°C until use.

### Western blotting analysis

MCF10A cells were washed with PBS and quickly trypsinized. The cells were lysed in radioimmunoprecipitation assay lysis buffer (RIPA lysis buffer) solution containing 50 mM Tris, 1% NP-40, 0.25% sodium deoxycholate, 0.1% SDS, 150 mM NaCl, 1 mM EDTA, 1 mM PMSF, and 1% protease inhibitor cocktail adjusted to pH 7.4 with HCl. The lysate was incubated on ice for 30 minutes and centrifuged for 20 minutes at 12,000 rpm and 4°C. The supernatant was then collected and stored at −20°C until use. The amount of soluble protein in the lysate was determined using BCA™ Protein Assay Kit (Pierce, USA). Next, 70 μg of proteins were loaded for the detection of Ku70, Ku80, *m*-calpain, histone, and β-actin, and 100 μg of proteins was loaded for the detection of γ-H2AX. Samples were run on 10-15% sodium dodecyl sulfate polyacrylamide gel electrophoresis (SDS-PAGE) and transferred to a 0.2 μm polyvinylidene fluoride membrane (Millipore Corporation, USA) for the detection of γ-H2AX and a 0.45 μm membrane for the detection of the remaining proteins at 200 mA for 1 to 2 hours. The membranes were subsequently blocked with 5% skimmed milk in Tris-buffered saline containing 0.1% Tween 20 (TBST) for 1 hour at room temperature. After washing the membranes three times with TBST once every 20 minutes, they were incubated with primary antibodies against β-actin (loading control), Ku70, Ku80, *m*-calpain, and γ-H2AX in TBST with 5% bovine serum albumin (BSA) and histone (loading control) in TBST with 5% skimmed milk at 4°C overnight. The following day, the membranes were washed three times with TBST every 20 minutes, and secondary antibodies of anti-mouse IgG horseradish peroxidase for histones and anti-rabbit IgG horseradish peroxidase for the remaining proteins were incubated for 2 hours at room temperature. Membranes were again washed three times with TBST every 20 minutes and detected with Power Opti-ECL Western blotting detection reagent (Animal Genetics Inc., Korea) and West-Q Femto Clean ECL solution reagent (GenDEPOT, USA). Western blot images were produced using a LAS-3000 and ChemiDoc™ XRS and analyzed using Multi-Gauge Software (Fuji Photo Film Co. Ltd., Japan) and Image Lab™ Software (Biorad, USA), respectively. Primary antibodies against Ku70, Ku80, and histone were from Santa Cruz Biotech (CA), that against γ-H2AX was from Abcam (USA), and those against *m*-calpain, β-actin, flag, and all secondary antibodies were from Cell Signaling (USA). All primary antibodies were diluted at a ratio of 1:1000, and secondary antibodies were diluted at a ratio of 1:2000.

### Two-dimensional (2D) electrophoresis and mass spectrometry

Nuclear extracts of MCF10A cells untreated or treated with 1 μM of ionomycin for 2 hours were prepared using the same process as used for cellular fractionation without the addition of buffer B. For electrophoresis, nuclear extracts were lysed with two-dimensional electrophoresis buffer containing 7 M urea, 2 M thiourea, 4% 3-[(3-cholamidopropyl)dimethylammonio]-1-propanesulfonate (CHAPS), and 2.5% DTT and sonicated for full lysis. Next, 700 μg of proteins of each prepared nuclear extract were loaded and electrophoresed using Immobiline™ DryStrip pH 3-10, 18 cm gels (GE healthcare Life Sciences, USA). After electrophoresis, gels were stained with Coomassie Blue G-250. 2DE gels and mass spectrometry for the prepared nuclear extracts were performed by ProteomeTech (Korea).

### Immunofluorescence assay

MCF10A cells were seeded at a density of 10^5^ cells per well in an 8-well cell culture slide (SPL, Korea). The following day, the cells were incubated with ionomycin in serum-free medium for 2 hours. After being washed with ice-cold PBS, cells were then fixed with 3.7% formaldehyde in PBS and incubated at 37°C for 20 minutes. The fixed cells were washed three times with PBS and permeated by incubation at room temperature for 10 minutes with immunofluorescence lysis buffer containing 20 mM HEPES, 50 mM NaCl, 3 mM MgCl_2_, 300 mM sucrose, and 0.5% Triton X-100. After washing with PBS, the cell culture slide was incubated with blocking solution containing 3% BSA in PBS for 1 hour at room temperature and subsequently with anti-*m*-calpain antibodies in blocking solution overnight at 4°C. The following day, the cell culture slide was washed three times with PBS every 10 minutes and incubated with an AlexaFluor488-conjugated secondary antibody (Cell Signaling, USA) in blocking solution for 2 hours. After washing three times with PBS every 10 minutes, cells were incubated with 4^′^-6^'^-diamidino-2-phenylindole (DAPI) for 30 minutes at 4°C in the dark, and coverslips (Deckglaser, Germany) were mounted with fluorescence mounting medium (Dako, Denmark) and sealed with clear nail polish. Images were captured using a Zeiss LSM510 META confocal microscope (Carl Zeiss, Germany) and merged using a Carl Zeiss imaging system.

### Lentivirus-mediated small hairpin RNA interference

MCF10A cells were seeded in 6-well plates (SPL, Korea) and incubated until the confluency was greater than 90%. Subsequently, 1 mL of culture medium was removed, and 1 mL of shRNA lentivirus and 8 μg/mL of polybrene were added. *m*-Calpain-targeting shRNAs in four different pLKO.1-shRNA plasmids were kindly provided by Prof. H.G. Yoon (Yonsei Univ, Korea). After 2 days, cells were moved to a 100-mm cell culture plate (Nunc, USA), and shRNA-infected cells were selected with 3 μg/mL puromycin. The surviving cells were maintained for 2 weeks with 1 μg/mL puromycin, and the expression level of *m*-calpain was monitored by Western blotting analysis.

### *In vitro* μ- and *m*-calpain assay

The *in vitro μ*- and *m*-calpain assay was performed by mixing each protein in μ- and *m*-calpain reaction buffer, respectively. *μ*-Calpain reaction buffer was composed of 50 mM Tris-HCl, 50 mM NaCl, 1 mM EDTA, 1 mM EGTA, and 5 mM β-mercaptoethanol adjusted to pH 7.5 with HCl, and *m*-calpain reaction buffer contained 50 mM Tris-HCl, 100 mM NaCl, 5 mM β-mercaptoethanol, and 1 mM EDTA, with pH adjusted to 7.5 with HCl. Ku70 and Ku80 proteins were purchased from Abcam (USA). *μ*-Calpain (human erythrocyte) and *m*-calpain (recombinant, High Purity, *E. Coli*) enzymes were obtained from Calbiochem (Germany). Then, 0.2 μg of Ku proteins and 0.8 units of each calpain were mixed in the respective calpain buffer, and calcium was added. The final concentration of calcium was 2.5 mM and 5 mM for μ- and *m*-calpain activation, respectively, according to previous studies [69, 70]. The reaction was stopped by the addition of 5 mM EDTA and 2X sample buffer (125 mM Tris-HCl, 20% glycerol, 4% SDS, 10% β-mercaptoethanol, and 0.6 μM bromophenol blue), and Western blotting analysis was performed.

### Transfection

In order to identify the cleavage domain of Ku80, genes encoding the amino acids 1-430 of the N-terminus of Ku80 and the amino acids 431-732 of the C-terminus of the Ku 80 were used, a generous gift from Prof. Y.S. Lee (Ewha Womans Univ. Korea). To transfect each gene, 239FT cells were seeded in 60-mm cell culture plates at a density of 5 × 10^5^ cells and incubated for various times using the WelFect QTM Plus kit (Welgene, Korea). The most efficient transfection time was elucidated by monitoring the extent of protein expression by Western blotting analysis using an anti-flag antibody (Cell Signaling, USA). Following transfection, cells were incubated with 0.75 μM of ionomycin for 2 hours. Results were obtained by Western blotting analysis.

### Immunoprecipitation assay

Immunoprecipitation was performed in 3 steps; preparation of the lysates, pre-clearing of the lysates, and precipitation of the immune complexes. Firstly, MCF10A cells were lysed with RIPA buffer, and lysate samples were prepared in the same process as for Western blotting analysis. Secondly, 500 μg of cell lysates were incubated with 1 μg of normal IgG (Santa Cruz Biotech, CA) and 1 μL of protein A-agarose beads (Santa Cruz Biotech, CA) at 4°C for 30 minutes with gentle shaking and then centrifuged at 4°C and 12000 rpm for 10 minutes. The supernatant was collected, and the anti-Ku70 antibody was added and incubated overnight at 4°C with rotation. The following day, the protein A-agarose beads were added, rocked for 2 hours, and centrifuged at 4°C and 12000 rpm for 10 minutes. The pellet was collected after washing twice with RIPA buffer. Immune complexes were resuspended in 20 μL 2X SDS sample buffer and boiled at 95°C for 5 minutes. Samples were separated by SDS-PAGE electrophoresis.

### Comet assay

The comet assay was performed using the Alkaline CometAssay^®^ kit (Trevigen, USA). MCF10A cells were seeded in 6-well cell culture plates at a density of 10^5^ cells per well and cultured for 2 days. Cells were incubated with or without 3 μM adriamycin, 0.75 μM ionomycin, and 50 μM calpeptin for 3 hours in serum-free media. Prior to harvesting cells, LMAgarose was melted in a beaker of boiling water and then cooled in a 37°C water bath. Trypsinized cells were combined with molten LMAgarose at a ratio of 1:10, immediately transferred onto a CometSlide™, and incubated at 4°C in the dark for 10 minutes. The slides were subsequently immersed in lysis solution that had been cooled to 4°C before use. After 30 minutes, the slides were incubated in alkaline unwinding solution (0.2 M NaOH, 1 mM EDTA) for 20 minutes at room temperature and electrophoresed at 15 V for 20 minutes in electrophoresis solution (0.2 M NaOH, 1 mM EDTA, pH>13). The slides were then washed twice with double distilled water and once with 70% ethanol for 5 minutes each. Subsequently, the samples were dried at 37°C for 2 hours and stained with diluted SYBR^®^ Green for 15 minutes in the dark, mounted on coverslips, and sealed with clear nail polish. Images were captured using a Zeiss HBO100 microscope illumination system (Carl Zeiss, Germany) equipped with an epq100-isolated epifluorescence condenser. Approximately 100 spots of MCF10A cells were randomly analyzed with an image analysis system (Komet 5.5, Kinetic Imaging Ltd, UK). The Komet 5.5 software calculated the lengths of the comet tails, and the mean values represent the extent of the DNA damage.

### Statistical analysis

All experiments were performed at least three times, and all data are expressed as the mean ± standard deviation. Statistics were calculated by one-way analysis of variance (ANOVA) using GraphPad Instat version 3.10 (GraphPad Software, USA), and the differences between two values were considered statistically significant when *p* values, described with single or double asterisks, were <0.05 and <0.01, respectively.

## SUPPLEMENTARY TABLES



## References

[1] Helleday T PE, Lundin C, Hodgson B, Sharma RA (2008). DNA repair pathways as targets for cancer therapy. Nature reviews Cancer.

[2] Liu PF, Chang WC, Wang YK, Munisamy SB, Hsu SH, Chang HY, Wu SH, Pan RL (2007). Differential regulation of Ku gene expression in etiolated mung bean hypocotyls by auxins. Biochimica et biophysica acta.

[3] Hsiang YH, Lihou MG, Liu LF (1989). Arrest of replication forks by drug-stabilized topoisomerase I-DNA cleavable complexes as a mechanism of cell killing by camptothecin. Cancer research.

[4] Markovits J, Pommier Y, Kerrigan D, Covey JM, Tilchen EJ, Kohn KW (1987). Topoisomerase II-mediated DNA breaks and cytotoxicity in relation to cell proliferation and the cell cycle in NIH 3T3 fibroblasts and L1210 leukemia cells. Cancer research.

[5] Hoeijmakers JH (2009). DNA damage, aging, and cancer. The New England journal of medicine.

[6] van Gent DC, Hoeijmakers JH, Kanaar R (2001). Chromosomal stability and the DNA double-stranded break connection. Nature reviews Genetics.

[7] Jackson SP (2002). Sensing and repairing DNA double-strand breaks. Carcinogenesis.

[8] van den Bosch M, Bree RT, Lowndes NF (2003). The MRN complex: coordinating and mediating the response to broken chromosomes. EMBO reports.

[9] Saleh-Gohari N, Helleday T (2004). Conservative homologous recombination preferentially repairs DNA double-strand breaks in the S phase of the cell cycle in human cells. Nucleic acids research.

[10] Rothkamm K, Kruger I, Thompson LH, Lobrich M (2003). Pathways of DNA double-strand break repair during the mammalian cell cycle. Molecular and cellular biology.

[11] Mao Z, Bozzella M, Seluanov A, Gorbunova V (2008). DNA repair by nonhomologous end joining and homologous recombination during cell cycle in human cells. Cell cycle.

[12] Verdun RE, Karlseder J (2006). The DNA damage machinery and homologous recombination pathway act consecutively to protect human telomeres. Cell.

[13] Burma S, Chen BP, Chen DJ (2006). Role of non-homologous end joining (NHEJ) in maintaining genomic integrity. DNA repair.

[14] Reynolds P, Anderson JA, Harper JV, Hill MA, Botchway SW, Parker AW, O'Neill P (2012). The dynamics of Ku70/80 and DNA-PKcs at DSBs induced by ionizing radiation is dependent on the complexity of damage. Nucleic acids research.

[15] Kato M, Iida S, Komatsu H, Ueda R (2002). Lack of Ku80 alteration in multiple myeloma. Japanese journal of cancer research.

[16] Rampakakis E, Di Paola D, Zannis-Hadjopoulos M (2008). Ku is involved in cell growth, DNA replication and G1-S transition. Journal of cell science.

[17] Gullo C, Au M, Feng G, Teoh G (2006). The biology of Ku and its potential oncogenic role in cancer. Biochimica et biophysica acta.

[18] Feldmann E, Schmiemann V, Goedecke W, Reichenberger S, Pfeiffer P (2000). DNA double-strand break repair in cell-free extracts from Ku80-deficient cells: implications for Ku serving as an alignment factor in non-homologous DNA end joining. Nucleic acids research.

[19] Nussenzweig A, Sokol K, Burgman P, Li L, Li GC Hypersensitivity of Ku80-deficient cell lines and mice to DNA damage: the effects of ionizing radiation on growth, survival, and development.

[20] Walker JR, Corpina RA, Goldberg J (2001). Structure of the Ku heterodimer bound to DNA and its implications for double-strand break repair. Nature.

[21] Wei F, Yan J, Tang D, Lin X, He L, Xie Y, Tao L, Wang S (2013). Inhibition of ERK activation enhances the repair of double-stranded breaks via non-homologous end joining by increasing DNA-PKcs activation. Biochimica et biophysica acta.

[22] Hawkins AJ, Golding SE, Khalil A, Valerie K (2011). DNA double-strand break - induced pro-survival signaling. Radiotherapy and oncology.

[23] Tremper-Wells B, Vallano ML (2005). Nuclear calpain regulates Ca2+-dependent signaling via proteolysis of nuclear Ca2+/calmodulin-dependent protein kinase type IV in cultured neurons. The Journal of biological chemistry.

[24] Chou SM, Huang TH, Chen HC, Li TK (2011). Calcium-induced cleavage of DNA topoisomerase I involves the cytoplasmic-nuclear shuttling of calpain 2. Cellular and molecular life sciences.

[25] Hizaki K, Yamamoto H, Taniguchi H, Adachi Y, Nakazawa M, Tanuma T, Kato N, Sukawa Y, Sanchez JV, Suzuki H, Sasaki S, Imai K, Shinomura Y (2011). Epigenetic inactivation of calcium-sensing receptor in colorectal carcinogenesis. Modern pathology.

[26] Sergeev IN (2012). Vitamin D and cellular Ca2+ signaling in breast cancer. Anticancer research.

[27] Lakshmikuttyamma A, Selvakumar P, Kanthan R, Kanthan SC, Sharma RK (2004). Overexpression of m-calpain in human colorectal adenocarcinomas. Cancer epidemiology, biomarkers & prevention.

[28] Storr SJ, Lee KW, Woolston CM, Safuan S, Green AR, Macmillan RD, Benhasouna A, Parr T, Ellis IO, Martin SG (2012). Calpain system protein expression in basal-like and triple-negative invasive breast cancer. Annals of oncology.

[29] Storr SJ, Carragher NO, Frame MC, Parr T, Martin SG (2011). The calpain system and cancer. Nature reviews Cancer.

[30] Goll DE, Thompson VF, Li H, Wei W, Cong J (2003). The calpain system. Physiological reviews.

[31] Leloup L WA (2011). Calpains as potential anti-cancer targets. Expert opinion on therapeutic targets.

[32] Xie M, Kobayashi I, Kiyoshima T, Yamaza H, Honda JY, Takahashi K, Enoki N, Akamine A, Sakai H (2007). Functional implication of nucleolin in the mouse first molar development. The Journal of biological chemistry.

[33] Wang KK VA, Roufogalis BD (1989). Calmodulin-binding proteins as calpain substrates. The Biochemical journal.

[34] Kimura E, Abe K, Suzuki K, Sorimachi H (2003). Heterogeneous nuclear ribonucleoprotein K interacts with and is proteolyzed by calpain in vivo. Bioscience, biotechnology, and biochemistry.

[35] Liu X, Van Vleet T, Schnellmann RG (2004). The role of calpain in oncotic cell death. Annual review of pharmacology and toxicology.

[36] Franco S, Perrin B, Huttenlocher A (2004). Isoform specific function of calpain 2 in regulating membrane protrusion. Experimental cell research.

[37] Taylor RG, Geesink GH, Thompson VF, Koohmaraie M, Goll DE (1995). Is Z-disk degradation responsible for postmortem tenderization?. Journal of animal science.

[38] Santella L, Kyozuka K, Hoving S, Munchbach M, Quadroni M, Dainese P, Zamparelli C, James P, Carafoli E (2000). Breakdown of cytoskeletal proteins during meiosis of starfish oocytes and proteolysis induced by calpain. Experimental cell research.

[39] Shaw G, Yang C, Zhang L, Cook P, Pike B, Hill WD (2004). Characterization of the bovine neurofilament NF-M protein and cDNA sequence, and identification of in vitro and in vivo calpain cleavage sites. Biochemical and biophysical research communications.

[40] Chan SO, Runko E, Anyane-Yeboa K, Ko L, Chiu FC (1998). Calcium ionophore-induced degradation of neurofilament and cell death in MSN neuroblastoma cells. Neurochemical research.

[41] Nelson WJ, Traub P (1983). Proteolysis of vimentin and desmin by the Ca2+-activated proteinase specific for these intermediate filament proteins. Molecular and cellular biology.

[42] Mori K, Muto Y, Kokuzawa J, Yoshioka T, Yoshimura S, Iwama T, Okano Y, Sakai N (2004). Neuronal protein NP25 interacts with F-actin. Neuroscience research.

[43] Pilop C, Aregger F, Gorman RC, Brunisholz R, Gerrits B, Schaffner T, Gorman JH, Matyas G, Carrel T, Frey BM (2009). Proteomic analysis in aortic media of patients with Marfan syndrome reveals increased activity of calpain 2 in aortic aneurysms. Circulation.

[44] Covault J, Liu QY, el-Deeb S (1991). Calcium-activated proteolysis of intracellular domains in the cell adhesion molecules NCAM and N-cadherin. Brain research Molecular brain research.

[45] Libertini SJ, Robinson BS, Dhillon NK, Glick D, George M, Dandekar S, Gregg JP, Sawai E, Mudryj M (2005). Cyclin E both regulates and is regulated by calpain 2, a protease associated with metastatic breast cancer phenotype. Cancer research.

[46] Bevers MB, Lawrence E, Maronski M, Starr N, Amesquita M, Neumar RW (2009). Knockdown of m-calpain increases survival of primary hippocampal neurons following NMDA excitotoxicity. Journal of neurochemistry.

[47] Gao G, Dou QP (2000). N-terminal cleavage of bax by calpain generates a potent proapoptotic 18-kDa fragment that promotes bcl-2-independent cytochrome C release and apoptotic cell death. Journal of cellular biochemistry.

[48] Mandic A, Viktorsson K, Strandberg L, Heiden T, Hansson J, Linder S, Shoshan MC (2002). Calpain-mediated Bid cleavage and calpain-independent Bak modulation: two separate pathways in cisplatin-induced apoptosis. Molecular and cellular biology.

[49] Wood DE, Newcomb EW (2000). Cleavage of Bax enhances its cell death function. Experimental cell research.

[50] Nakagawa T, Yuan J (2000). Cross-talk between two cysteine protease families. Activation of caspase-12 by calpain in apoptosis. The Journal of cell biology.

[51] Schmaier AH, Smith PM, Purdon AD, White JG, Colman RW (1986). High molecular weight kininogen: localization in the unstimulated and activated platelet and activation by a platelet calpain(s). Blood.

[52] Xu L, Deng X (2006). Protein kinase Ciota promotes nicotine-induced migration and invasion of cancer cells via phosphorylation of micro- and m-calpains. The Journal of biological chemistry.

[53] Mamoune A, Luo JH, Lauffenburger DA, Wells A (2003). Calpain-2 as a target for limiting prostate cancer invasion. Cancer research.

[54] Su Y, Cui Z, Li Z, Block ER (2006). Calpain-2 regulation of VEGF-mediated angiogenesis. FASEB journal.

[55] Xu L, Deng X (2004). Tobacco-specific nitrosamine 4-(methylnitrosamino)-1-(3-pyridyl)-1-butanone induces phosphorylation of mu- and m-calpain in association with increased secretion, cell migration, and invasion. The Journal of biological chemistry.

[56] Gullo CA, Ge F, Cow G, Teoh G (2008). Ku86 exists as both a full-length and a protease-sensitive natural variant in multiple myeloma cells. Cancer cell international.

[57] Song JY, Lim JW, Kim H, Morio T, Kim KH (2003). Oxidative stress induces nuclear loss of DNA repair proteins Ku70 and Ku80 and apoptosis in pancreatic acinar AR42J cells. The Journal of biological chemistry.

[58] Mehdi S, Angelastro MR, Wiseman JS, Bey P (1988). Inhibition of the proteolysis of rat erythrocyte membrane proteins by a synthetic inhibitor of calpain. Biochem. Biophys. Res. Commun.

[59] Ivashkevich A, Redon CE, Nakamura AJ, Martin RF, Martin OA (2012). Use of the gamma-H2AX assay to monitor DNA damage and repair in translational cancer research. Cancer letters.

[60] Liao W, McNutt MA, Zhu WG (2009). The comet assay: a sensitive method for detecting DNA damage in individual cells. Methods.

[61] Donkor IO (2011). Calpain inhibitors: a survey of compounds reported in the patent and scientific literature. Expert opinion on therapeutic patents.

[62] Kato M, Nonaka T, Imajoh-Ohmi S (2005). Cleavage at the carboxyl-terminus of Ku80 during apoptosis in human Jurkat T cells. Journal of Biochemistry.

[63] Gell D, Jackson SP (1999). Mapping of protein-protein interactions within the DNA-dependent protein kinase complex. Nucleic Acids Research.

[64] Song JY, Lim JW, Kim H, Morio T, Kim KH (2003). Oxidative stress induces nuclear loss of DNA repair proteins Ku70 and Ku80 and apoptosis in pancreatic acinar AR42J cells. Journal of Biological Chemistry.

[65] Vos SM, Tretter EM, Schmidt BH, Berger JM (2011). All tangled up: how cells direct, manage and exploit topoisomerase function. Nature reviews Molecular cell biology.

[66] Kim SY, Kim SJ, Kim BJ, Rah SY, Chung SM, Im MJ, Kim UH (2006). Doxorubicin-induced reactive oxygen species generation and intracellular Ca2+ increase are reciprocally modulated in rat cardiomyocytes. Experimental & molecular medicine.

[67] Nair S, Samy TS, Krishan A (1986). Calcium, calmodulin, and protein content of adriamycin-resistant and -sensitive murine leukemic cells. Cancer research.

[68] Mircheva J, Tsuruo T (1990). Enhancement of adriamycin cytotoxicity in sensitive and resistant sublines of human tumor cells by calcium antagonists. Tumori.

[69] Lee E, Eom JE, Kim HL, Baek KH, Jun KY, Kim HJ, Lee M, Mook-Jung I, Kwon Y (2013). effect of conjugated linoleic acid, μ-calpain inhibitor, on pathogenesis of Alzheimer's disease. Biochim Biophys Acta.

[70] Kang DH, Jun KY, Lee JP, Pak CS, Na Y, Kwon Y (2009). Identification of 3-acetyl-2-aminoquinolin-4-one as a novel, nonpeptidic scaffold for specific calpain inhibitory activity. J Med Chem.

