# Supplementary material for: Calcium influx-mediated translocation of m-calpain induces Ku80 cleavage and enhances the Ku80-related DNA repair pathway
Source: Oncotarget. 2016 Apr 18;7(21):30831–44. doi: 10.18632/oncotarget.8791 (PMC5058721; doi:10.18632/oncotarget.8791)
Supplement: Supplementary file 1 [file oncotarget-07-30831-s001.pdf]

Calcium influx-mediated translocation of *m*-calpain induces Ku80 cleavage and enhances the Ku80-related DNA repair pathway

SUPPLEMENTARY DATA

REFERENCES

1. Liu X, Schnellmann RG. Calpain mediates progressive plasma membrane permeability and proteolysis of cytoskeleton-associated paxillin, talin, and vinculin during renal cell death. The Journal of pharmacology and experimental therapeutics. 2003; 304:63-70.
2. Taylor RG, Geesink GH, Thompson VF, Koohmaraie M, Goll DE. Is Z-disk degradation responsible for postmortem tenderization? Journal of animal science. 1995; 73:1351-1367.
3. Nath R, Raser KJ, Stafford D, Hajimohammadreza I, Posner A, Allen H, Talanian RV, Yuen P, Gilbertsen RB, Wang KK. Non-erythroid alpha-spectrin breakdown by calpain and interleukin 1 beta-converting-enzyme-like protease(s) in apoptotic cells: contributory roles of both protease families in neuronal apoptosis. The Biochemical journal. 1996; 319:683-690.
4. Chan SO, Runko E, Anyane-Yeboa K, Ko L, Chiu FC. Calcium ionophore-induced degradation of neurofilament and cell death in MSN neuroblastoma cells. Neurochemical research. 1998; 23:393-400.
5. Nelson WJ, Traub P. Proteolysis of vimentin and desmin by the Ca2+-activated proteinase specific for these intermediate filament proteins. Molecular and cellular biology. 1983; 3:1146-1156.
6. Pilop C, Aregger F, Gorman RC, Brunisholz R, Gerrits B, Schaffner T, Gorman JH, 3rd, Matyas G, Carrel T, Frey BM. Proteomic analysis in aortic media of patients with Marfan syndrome reveals increased activity of calpain 2 in aortic aneurysms. Circulation. 2009; 120:983-991.
7. Cortesio CL, Perrin BJ, Bennin DA, Huttenlocher A. Actin-binding protein-1 interacts with WASp-interacting protein to regulate growth factor-induced dorsal ruffle formation. Molecular biology of the cell. 2010; 21:186-197.
8. Cortesio CL, Perrin BJ, Bennin DA, Huttenlocher A. Actin-binding protein-1 interacts with WASp-interacting protein to regulate growth factor-induced dorsal ruffle formation. Molecular biology of the cell. 2010; 21:186-197.
9. Harada K, Fukuda S, Kunimoto M, Yoshida K. Distribution of ankyrin isoforms and their proteolysis after ischemia and reperfusion in rat brain. Journal of neurochemistry. 1997; 69:371-376.
10. Elamrani N, Balcerzak D, Soriano M, Brustis JJ, Cottin P, Poussard S, Ducastaing A. Evidence for fibronectin degradation by calpain II. Biochimie. 1993; 75:849-853.
11. Covault J, Liu QY, el-Deeb S. Calcium-activated proteolysis of intracellular domains in the cell adhesion molecules NCAM and N-cadherin. Brain research Molecular brain research. 1991; 11:11-16.
12. Yoshimoto R, Hori M, Ozaki H, Karaki H. Proteolysis of acidic calponin by mu-calpain. Journal of biochemistry. 2000; 128:1045-1049.
13. Cooray P, Yuan Y, Schoenwaelder SM, Mitchell CA, Salem HH, Jackson SP. Focal adhesion kinase (pp125FAK) cleavage and regulation by calpain. The Biochemical journal. 1996; 318:41-47.
14. Nakagawa T, Yuan J. Cross-talk between two cysteine protease families. Activation of caspase-12 by calpain in apoptosis. The Journal of cell biology. 2000; 150:887-894.
15. Blomgren K, Zhu C, Wang X, Karlsson JO, Leverin AL, Bahr BA, Mallard C, Hagberg H. Synergistic activation of caspase-3 by m-calpain after neonatal hypoxia-ischemia: a mechanism of “pathological apoptosis”? The Journal of biological chemistry. 2001; 276:10191-10198.
16. Bizat N, Hermel JM, Humbert S, Jacquard C, Creminon C, Escartin C, Saudou F, Krajewski S, Hantraye P, Brouillet E. In vivo calpain/caspase cross-talk during 3-nitropropionic acid-induced striatal degeneration: implication of a calpain-mediated cleavage of active caspase-3. The Journal of biological chemistry. 2003; 278:43245-43253.
17. Bevers MB, Lawrence E, Maronski M, Starr N, Amesquita M, Neumar RW. Knockdown of m-calpain increases survival of primary hippocampal neurons following NMDA excitotoxicity. Journal of neurochemistry. 2009; 108:1237-1250.
18. Cook JC, Chock PB. Isoforms of mammalian ubiquitin-activating enzyme. The Journal of biological chemistry. 1992; 267:24315-24321.
19. Fuchs SY, Spiegelman VS, Kumar KG. The many faces of beta-TrCP E3 ubiquitin ligases: reflections in the magic mirror of cancer. Oncogene. 2004; 23:2028-2036.
20. Gao G, Dou QP. N-terminal cleavage of bax by calpain generates a potent proapoptotic 18-kDa fragment that promotes bcl-2-independent cytochrome C release and apoptotic cell death. Journal of cellular biochemistry. 2000; 80:53-72.

21. Shimizu S, Konishi A, Kodama T, Tsujimoto Y. BH4 domain of antiapoptotic Bcl-2 family members closes voltage-dependent anion channel and inhibits apoptotic mitochondrial changes and cell death. Proceedings of the National Academy of Sciences of the United States of America. 2000; 97:3100-3105.
22. Wood DE, Newcomb EW. Cleavage of Bax enhances its cell death function. Experimental cell research. 2000; 256:375-382.
23. Chen M, He H, Zhan S, Krajewski S, Reed JC, Gottlieb RA. Bid is cleaved by calpain to an active fragment in vitro and during myocardial ischemia/reperfusion. The Journal of biological chemistry. 2001; 276:30724-30728.
24. Liu W, Zhou XW, Liu S, Hu K, Wang C, He Q, Li M. Calpain-truncated CRMP-3 and -4 contribute to potassium deprivation-induced apoptosis of cerebellar granule neurons. Proteomics. 2009; 9:3712-3728.
25. Kishimoto A, Mikawa K, Hashimoto K, Yasuda I, Tanaka S, Tominaga M, Kuroda T, Nishizuka Y. Limited proteolysis of protein kinase C subspecies by calcium-dependent neutral protease (calpain). The Journal of biological chemistry. 1989; 264:4088-4092.
26. Kakkar R, Raju RV, Sharma RK. Calmodulin-dependent cyclic nucleotide phosphodiesterase (PDE1). Cellular and molecular life sciences : CMLS. 1999; 55(8-9):1164-1186.
27. Samanta K, Kar P, Chakraborti T, Shaikh S, Chakraborti S. Characteristic properties of endoplasmic reticulum membrane m-calpain, calpastatin and lumen m-calpain: a comparative study between membrane and lumen m-calpains. Journal of biochemistry. 2010; 147:765-779.
28. Wang KK VA, Roufogalis BD. Calmodulin-binding proteins as calpain substrates. The Biochemical journal. 1989; 262:693.
29. Xie M, Kobayashi I, Kiyoshima T, Yamaza H, Honda JY, Takahashi K, Enoki N, Akamine A, Sakai H. Functional implication of nucleolin in the mouse first molar development. The Journal of biological chemistry. 2007; 282:23275-23283.
30. Liu X, Van Vleet T, Schnellmann RG. The role of calpain in oncotic cell death. Annual review of pharmacology and toxicology. 2004; 44:349-370.
31. Lin YC, Brown K, Siebenlist U. Activation of NF-kappa B requires proteolysis of the inhibitor I kappa B-alpha: signal-induced phosphorylation of I kappa B-alpha alone does not release active NF-kappa B. Proceedings of the National Academy of Sciences of the United States of America. 1995; 92:552-556.
32. Paulhe F, Bogyo A, Chap H, Perret B, Racaud-Sultan C. Vascular smooth muscle cell spreading onto fibrinogen is regulated by calpains and phospholipase C. Biochemical and biophysical research communications. 2001; 288:875-881.
33. Schmaier AH, Smith PM, Purdon AD, White JG, Colman RW. High molecular weight kininogen: localization in the unstimulated and activated platelet and activation by a platelet calpain(s). Blood. 1986; 67:119-130.
34. Pontremoli S, Melloni E, Sparatore B, Michetti M, Horecker BL. A dual role for the Ca2+-requiring proteinase in the degradation of hemoglobin by erythrocyte membrane proteinases. Proceedings of the National Academy of Sciences of the United States of America. 1984; 81:6714-6717.
35. Bradford HN, Annamalai A, Doshi K, Colman RW. Factor V is activated and cleaved by platelet calpain: comparison with thrombin proteolysis. Blood. 1988; 71:388-394.
36. Moore JC, Murphy WG, Kelton JG. Calpain proteolysis of von Willebrand factor enhances its binding to platelet membrane glycoprotein IIb/IIIa: an explanation for platelet aggregation in thrombotic thrombocytopenic purpura. British journal of haematology. 1990; 74:457-464.

Supplementary Table S1: Calpain substrates among cytoskeletal and structural proteins

Substrate	Comments
Paxilin, Talin [1]	Increase plasma membrane permeability
Vinculin [2]	Membrane-cytoskeletal protein in focal adhesion plaques
α-spectrin [3]	Prognostic marker after traumatic brain injury<br>Three cleavage products; 150, 145, and 120 kDa.
Internexin [4]	Brain and central nervous system filament
Vimentin [5]	Filament protein. Degraded after cleavage
Desmin [5]	Subunit of intermediate filaments
NP-25 [6]	Involved in regulation of actin filament stability
Cortactin [7]	F-actin binding protein
mAbp1 [8] (mammalian actin-binding protein)	Cleavage site is located between the actin-binding domain and the proline-rich region<br>Inhibits dorsal ruffle formation after cleavage
Filamin A [6]	Also cleaved by caspase and granzyme
Ankyrin [9]	Maintains cellular integrity and shape
Fibronectin [10]	Related to cell adhesion, growth, migration, and differentiation
Cadherin [11]	Only N-cadherin is cleaved, not E-cadherin
Calponin [12]	Calmodulin and F-actin binding protein
Focal adhesion kinase [13]	Regulates adhesion dynamics.

Supplementary Table S2: Calpain substrates among apoptosis regulators and cell signaling proteins

Substrate	Comments
Caspase [14, 15, 16]	Cysteine-aspartic protease<br>Many isoforms such as caspase-3,7, and 12 are cleaved
AIF [17] (Apoptosis inducing factor)	Translocated from mitochondria to the nucleus after cleavage
β-TrCP [18, 19] (β-Transducin repeat containing protein)	Components of the ubiquitin-proteasome degradation system and regulator of cell cycle checkpoints<br>Being disrupted causing neuronal apoptosis after cleavage
Bcl-2 [20, 21], Bax [22], Bid [23]	Closely involved in apoptosis<br>Govern mitochondrial outer membrane permeabilization (MOMP)
E1 [24] (Ubiquitin-activating enzyme E1)	Components of the ubiquitin-proteasome degradation system<br>Cleavage product is approximately 105 kDa
PKC [25]	Performs multiple functions
Phosphodiesterase [26]	Activated after cleavage
Inositol 1,4,5-triphosphate receptor-1 [27]	Cleaved in ER lumen<br>Secondary messenger
Phosphorylase kinase [28]	Activated after cleavage

Supplementary Table S3: Calpain substrates among transcription factors and blood coagulation-related proteins

Group	Substrate	Comments
Transcription factors	Nucleolin [29]	Leads to apoptosis by reducing RNA synthesis and processing
	Hnrp K / F [28, 30]	Heterogeneous nuclear ribonucleoprotein K / F
	IkBα [31]	Blocks the ability of NF-κB
	Histone 2A, B [28]	Compact DNA strands
Blood coagulation related proteins	Fibrinogen [32]	Soluble plasma glycoprotein
	Kininogen [33]	Increases coagulant activity after proteolysis
	Haemoglobin [34]	Oxygen-transport metalloprotein
	Factor V [35]	Functions as a cofactor in the coagulation system
	Von Willebrand factor [36]	Blood glycoprotein involved in hemostasis
